# Evaluation of template‐based modeling in CASP13

**DOI:** 10.1002/prot.25800

**Published:** 2019-08-20

**Authors:** Tristan I. Croll, Massimo D. Sammito, Andriy Kryshtafovych, Randy J. Read

**Affiliations:** ^1^ Department of Haematology University of Cambridge, Cambridge Institute for Medical Research Cambridge UK; ^2^ Genome Center University of California Davis California

**Keywords:** CASP, molecular replacement, structure prediction, template‐based modeling

## Abstract

Performance in the template‐based modeling (TBM) category of CASP13 is assessed here, using a variety of metrics. Performance of the predictor groups that participated is ranked using the primary ranking score that was developed by the assessors for CASP12. This reveals that the best results are obtained by groups that include contact predictions or inter‐residue distance predictions derived from deep multiple sequence alignments. In cases where there is a good homolog in the wwPDB (TBM‐easy category), the best results are obtained by modifying a template. However, for cases with poorer homologs (TBM‐hard), very good results can be obtained without using an explicit template, by deep learning algorithms trained on the wwPDB. Alternative metrics are introduced, to allow testing of aspects of structural models that are not addressed by traditional CASP metrics. These include comparisons to the main‐chain and side‐chain torsion angles of the target, and the utility of models for solving crystal structures by the molecular replacement method. The alternative metrics are poorly correlated with the traditional metrics, and it is proposed that modeling has reached a sufficient level of maturity that the best models should be expected to satisfy this wider range of criteria.

## 1 INTRODUCTION

Even though the worldwide Protein Data Bank^1^ (wwPDB) continues to expand quickly, growth in this database is outpaced by the growth in genomic information, leading to an escalation in the need for protein structure modeling. Around the turn of the century the wwPDB archive was doubling every 3 to 4 years with apparent exponential growth, but in recent years it has taken about 7 years to double^2^; in contrast, genome databases are currently doubling about once every 7 months.^3^

A large fraction of proteins lacking an experimental structure will be at least distantly related to a protein of known structure, which can serve as a template for modeling. Template‐based modeling (TBM) plays a key role in leveraging genomic data—but as the level of sequence identity drops, TBM becomes progressively more challenging. In the early days of the CASP experiments, it is probably fair to say that many attempts to improve on the best template actually turned it into a worse model. However, great advances have been made over the years, aided in part by improved understanding of the energetics of protein folding^4^ but also largely by taking advantage of the growing databases. In CASP12, substantial improvements over CASP11 were attributed to several factors: better use of multiple templates; improved model refinement methods; and better methods for estimating model accuracy, which allowed the best alternative model to be chosen and focus limited computational resources on regions to refine.^5^ It was of interest to see whether CASP13 would reveal continued progress and, if so, what was driving it.

We have also taken the opportunity to look at some less conventional measures of quality. The traditional scoring metrics are defined primarily based on the deviation between model and target in Cartesian space, and for historical reasons are somewhat lenient—in early CASP rounds simply getting most Cα positions reasonably close to the target was a substantial achievement.^6^, ^7^ However, as the field matures the number of groups achieving high scores on any given model is steadily increasing. It is sensible to start considering more stringent measures of model quality, preferably orthogonal to those in current use. Thus, we have developed some measures based on how well the torsion angles describing the conformation of the structure are reproduced in the model. While torsion‐based analyses have been previously used in assessing CASP rounds 3,^8^ 4^9^ and 9^10^ they have not been widely adopted—perhaps because such metrics only become truly meaningful once the majority of the fold is essentially correct.

The conventional metrics, and the new torsion metrics, evaluate respectively the correctness of the predicted folds and the adherence of predicted fine‐scale features to those observed in the target structures. However, users of such models will primarily be interested in their utility for particular purposes, such as providing targets for the design of new therapeutics or explaining the impact of mutations found in inherited diseases. In addition to such applied research, predicted structures can also be very useful as initial models when determining new experimental structures. Arguably, the most common example of this is in X‐ray crystallography structure phasing through molecular replacement (MR).^11^ In MR, an atomic model derived from a related protein structure is rotated and translated in a search for the position occupied by the true structure in the crystal; phases calculated from the model are combined with data to produce an electron density map that reveals new features, if the model is sufficiently accurate. MR, when it succeeds, allows a structure to be determined from a data set from a single native crystal, without requiring the preparation of heavy‐atom derivatives or the accurate measurement of anomalous scattering data.^12^ TBM methods that improve significantly on the original template can therefore shortcut the process of structure determination and improve throughput in X‐ray crystallography. In recognition of this, in CASP7 we introduced a metric scoring individual model predictions based on their usefulness in MR.^6^ As a result of continuing improvement in structure prediction, the use of TBM to improve MR models has been greatly expanding in recent years.^13^, ^14^, ^15^, ^16^ Although TBM is the focus of this work, it should also be noted that free modeling of whole proteins or fragments can also yield useful models for MR, under favorable circumstances of relatively small proteins and high‐resolution data.^17^, ^18^, ^19^, ^20^

## 2 MATERIALS AND METHODS

### 2.1 Target classification and scope of this work

Target classification is described in detail elsewhere in this volume. Briefly, in CASP13 as in earlier exercises, targets for structural modeling were divided when appropriate into evaluation units, which were categorized by difficulty. The difficulty category addressed here, TBM, broadly covers cases in which a good template can be found in the PDB. It is further subdivided into TBM‐easy and TBM‐hard. For the most part, we will not be discussing the targets lacking good templates, which are categorized as free modeling (FM) or, for borderline cases, TBM/FM. For CASP13, there were 40 evaluation units defined as TBM‐easy and 21 defined as TBM‐hard.

### 2.2 Traditional evaluation measures

Over the years, a large number of evaluation measures have been developed to assess different aspects of model quality. A detailed description, classification and review of a number of these metrics has been published recently^21^; they differ for instance in whether or not they depend on structure superposition and whether they depend on global or local measures. Most of these metrics are computed, collated, and analyzed by the Prediction Center (http://predictioncenter.org),^22^ making them much more convenient for assessors and others.

In this work, our primary ranking has adopted the same overall ranking score used for TBM models in CASP12,^5^ which is based on five metrics computed by the Prediction Center. GDT_HA is the high‐accuracy version of the Global Distance Test, which assesses the overall fold in a way that gives greater reward for parts of the target reproduced with high precision.^23^ The local difference distance test, lDDT, evaluates how well models reproduce an all‐atom distance map.^7^ The contact area difference score, CADaa, is based on comparing residue contact surface areas.^24^ Sphere‐Grinder (SG) measures how well local environment is conserved between the model and target.^25^ Finally, the accuracy self‐estimate measure, ASE, evaluates the degree to which the coordinate error estimates predict positional differences from the target.^22^ The overall ranking score combining these measures is given by the following:

$$S_{\text{CASP}12}=\frac{1}{3}z_{\mathrm{GDT}\_\mathrm{HA}}+\frac{1}{9}\left(z_{\text{lDDT}}+z_{\text{CADaa}}+z_{\mathrm{SG}}\right)+\frac{1}{3}z_{\mathrm{ASE}}$$

This scheme assigns equal overall weight to global fold quality (GDT_HA), local structure quality (split over lDDT, CADaa, and SG), and quality of model accuracy estimates (ASE). In the ranking equation, *z* indicates the adjusted *z*‐score over all models under consideration for a given target, with the subscript denoting the particular underlying evaluation measure. The adjusted *z*‐score (essentially the number of standard deviations (SD) above the mean of the full set of models) is computed using the following protocol common to recent CASPs. A set of initial *z*‐scores is evaluated based on the mean and SD of scores from all the models under consideration. All models yielding initial *z*‐scores below −2 are omitted as potential outliers and the *z*‐scores are recomputed using the mean and SD from the pruned set of models. Finally, negative *z*‐scores are reset to zero, with the goal of reducing the penalty on predictors who test novel methods.

When comparing with other metrics it was sometimes more sensible to exclude the ASE component, leading to:

$$S_{\text{CASP}12-\mathrm{ASE}}=\frac{1}{2}z_{\mathrm{GDT}\_\mathrm{HA}}+\frac{1}{6}\left(z_{\text{lDDT}}+z_{\text{CADaa}}+z_{\mathrm{SG}}\right)$$

### 2.3 Geometric model quality metrics

Note: the geometric scoring functions we used were changed in response to suggestions from a reviewer. Our manuscript on assessment of refinement (Read et al, this issue) made use of the original functions and referred to this manuscript for their definitions. We have therefore provided the definitions for the original scoring functions in the Supplementary Information. The effects of the change are quite modest, with some reordering of closely spaced scores.

Here, we have implemented new metrics measuring the correspondence of predicted torsion angles to the target, separately evaluating local backbone and sidechain conformations, and applied them to compare both model and template (where a template was named) to the target structure. The score for a given dihedral was defined based on the metric previously used for protein dihedral analysis by North et al^26^ (the squared length of the chord on a unit circle resulting from the angular deviation from the target), normalized to the range (0…1):

$$\Gamma_{\text{torsion}}=\frac{1-\cos\left(\Delta \text{torsion}\right)}{2}$$

For each residue present in both model or template and target, the backbone score was defined as:

$$S_{\text{backbone}}=\frac{\Gamma_{\varphi }+\Gamma_{\psi }+\Gamma_{\omega }}{3}$$

where *φ* and *ψ* are the characteristic Ramachandran torsion angles^27^ and *ω* is the torsion across the peptide bond. Instances where the ω torsion was more than 30° from planar or flipped relative to the target (ie, *trans* to *cis* or vice versa) were separately recorded.

Devising a useful and fair score for sidechain conformations was somewhat more challenging. In experimental models, the sidechain conformation is often inherently less certain than the backbone, and in fact in highly solvent‐exposed locations there is often effectively no experimental evidence for any given configuration, and hence no true correct answer. Further, the certainty of a given sidechain torsion tends to reduce with distance from the backbone. To complicate matters further, the relevance of each torsion is dependent on those preceding: if the first torsion is completely wrong, then the values of the remaining torsion angles are effectively meaningless. The sidechain score for rotameric residues was thus defined as:

$$S_{\text{sidechain}}=\left\{\begin{array}{c} \beta \left(\Gamma_{\chi_{1}}\right)\;\text{if}\;n_{\chi }=1\\ \beta \left(1-\frac{2}{3}\left(1-\Gamma_{\chi_{1}}\right)-\frac{1}{3}e^{-{\left(\frac{{\Delta \chi }_{1}}{\tau }\right)}^{2}}\left(1-\Gamma_{\chi_{2}}\right)\right)\;\text{otherwise} \end{array}\right.$$

where *χ*_*i*_ is the *i*th sidechain torsion from the backbone, *n*_*χ*_ is the number of sidechain torsions in the given target residue, *τ* defines the contribution of *χ*_2_ to the score as a function of Δ*χ*_1_, and *β* is a “burial score” defined as:

$$\beta =\min\left(\frac{n_{\text{close}}}{3n_{\mathrm{sc}}},1\right)$$

where *n*_close_ is the number of heavy atoms from other residues within 4 å of any heavy atom in the given residue (based on the target structure), and *n*_sc_ is the number of heavy atoms expected in the sidechain. Sidechains with no *χ* torsions in the target (ie, glycine or alanine residues and truncations) did not receive a score. For any *χ* torsions present in the target but not in the model (eg, not modeled by the depositor, or mutated in the case of a template structure), Δ*χ* was given the maximum possible value of 180°. Any torsions present in the model but not in the target were ignored. We set the value of *τ* to 30°, such that the contribution of Γ*χ*_2_ becomes negligible when Δ*χ*_1_ > ≈50°. Values of the score range from 0 (Δ*χ*_1_ = Δ*χ*_2_ = 0) to 1 (Δ*χ*_1_ = 180°). A residue with *χ*_1_ perfectly matching the target and Δ*χ*_2_ = 180° would receive a score of 1/3.

For ranking of models, we found it convenient to define two different combined scores: a “torsion‐only” score, and a “geometry‐weighted” score combining torsion differences with more standard metrics. These are defined as:

$$S_{\text{torsion}}=\frac{2}{3}z_{\text{backbone}}+\frac{1}{3}z_{\text{sidechain}}$$

$$S_{\text{geom}}=\left(\begin{array}{c} \frac{1}{16}\left(z_{\text{lDDT}}+z_{\text{CADaa}}+z_{\mathrm{SG}}+z_{\text{sidechain}}\right)\\ +\frac{1}{8}\left(z_{\text{MolPrb}-\text{clash}}+z_{\text{backbone}}\right)\\ +\frac{1}{4}\left(z_{\mathrm{GDT}\_\mathrm{HA}}+z_{\mathrm{ASE}}\right) \end{array}\right)$$

where *z*‐scores were calculated as described in the previous section.

These analyses were implemented as Python scripts using tools from ChimeraX^28^ and ISOLDE.^29^

### 2.4 Evaluation by utility in molecular replacement calculations

In the high‐accuracy TBM assessment for CASP7, we tested each model for selected targets to determine whether it could have been used to solve the structure by molecular replacement.^6^ Model quality was measured by the log‐likelihood‐gain (LLG) score that measures agreement between atomic model and experimental diffraction data, for the best solution found by our likelihood‐based MR program *Phaser*.^30^ In order to keep the computing requirements within practical limits, targets were used and their submitted models were evaluated only if experimental diffraction data had been made available by the authors and there was only one copy of one component in the asymmetric unit of the crystal. To assess added value, the calculations were repeated using template structures available at the time of prediction in the PDB.

For this round, we used Python scripts prepared by Gábor Bunkóczi, subsequent to CASP7, for potential use in the CAMEO continuous automated model evaluation.^31^ In these scripts, a full MR search is not carried out; rather, the model is superimposed on each copy of the corresponding component in the crystal structure and subjected to rigid‐body refinement. This yields an appropriate LLG score regardless of whether the model is sufficiently accurate to succeed in the MR search. Because the LLG score becomes more sensitive as a model progressively becomes more complete, the test model is added to a background structure comprising the other components of the target structure, and the LLG score recorded is the difference between the LLG computed using the model and the LLG just with the background (if relevant). These changes to the LLG calculation allow us to use targets that are complexes or have multiple copies of molecules in the asymmetric unit, and the computing requirements are much less demanding than carrying out full MR searches. Nonetheless, a new understanding of the LLG score allows us to predict whether a full search would have succeeded, as searches that yield an increase in the LLG score of 60 units or more are almost always successful.^32^

Since CASP7, it has become much more common for predictors to submit local error estimates in the B‐factor fields of submitted models. As suggested at the time of the CASP7 evaluation,^6^ and as demonstrated in tests with models from CASP10,^33^ the use of error estimates to inflate local B‐factors of models, and thereby downweight the contributions of unreliable segments of the model at higher resolution, can dramatically increase the utility of models for MR. Increasing an atom's B‐factor by 8*π*^2^/3 times the square of the estimated RMS coordinate error has the effect of smearing the atom's electron density over its distribution of possible positions. To test the quality of error estimates, therefore, we evaluated models both using constant B‐factors and interpreting the B‐factor column as an RMS coordinate error estimate; as a control, a third calculation interpreted the B‐factor column as a B‐factor. The MR calculation is relatively inexpensive compared to modeling algorithms, and crystallographers tend to test multiple alternative models in practice. In accordance with this, we tested all five submitted models for each target and chose the best for each group.

To assess the value added from the modeling, we compared the results from the submitted models with what could have been achieved without modeling, using templates available in the PDB. For this comparison, we followed a protocol that would be recommended for users of *Phaser* (https://www.phaser.cimr.cam.ac.uk/index.php?Top_Ten_Tips). Sensitive sequence alignments, obtained at the time of prediction using HHpred,^34^ were downloaded from the Prediction Center and up to the five top hits (if significant at the level of *E*‐value < .0005) were tested as models, both as individual models and as ensembles. Models, pruned first to match the evaluation units, were prepared using the program Sculptor,^35^ which prunes atoms from loops and side chains that the sequence alignment implies are unlikely to be present in the target. Ensembles were prepared by superimposing models with the program Ensembler,^36^ activating the option to trim parts of the ensemble that are not conserved among the different ensemble members.

Preliminary trials indicated that the calculations could be unstable for models that reproduced the structure very poorly or when the coordinate error estimates were infeasibly large. For this reason, models with a GDT_TS score less than 30 or with a median coordinate error estimate greater than 3 å were omitted from the calculations and assigned an LLG score of zero.

An additional complication was encountered for two of the targets for which diffraction data were available, T0960 and T0963. The crystal structures for these targets display translational noncrystallographic symmetry (tNCS), in which more than one unique copy of a molecule is found in a similar orientation in the crystal. The presence of tNCS leads to a systematic modulation of the diffraction intensities, as the contributions from the tNCS‐related molecules can interfere constructively or destructively. If not accounted for, this seriously degrades the reliability of the MR calculations. *Phaser* has been adapted to characterize and account for the effects of tNCS,^37^ but because the Python scripts we were using have not yet been updated to take advantage of this new feature, we omitted these two targets from our calculations.

### 2.5 Model visualization

Models were visualized in ChimeraX,^28^ using validation markup provided by ISOLDE.^29^

## 3 RESULTS

### 3.1 Assessment of progress

As noted in previous CASP rounds it is difficult to assess progress, in no small part because the targets are different ones each time. This will add random noise to any comparison, but there are potential systematic effects as well. Most notably, the operational definition of an evaluation unit has a subjective element that could possibly mask some of the improvements that are being made: one of the considerations in defining an evaluation unit is whether or not any predictors succeeded in finding a good relative orientation between two segments of structure that might otherwise have been classified as separate domains. As predictors improve in this aspect, evaluation units may thus tend to become larger and more complex. In addition, the target structures since CASP11 have tended to be substantially larger; CASP13 even includes a few extremely large structures determined by cryo‐EM. This means that predictions of evaluation units might have become more difficult because there are more unknown environmental influences of neighboring parts of the complex structure.

**Figure 1:**
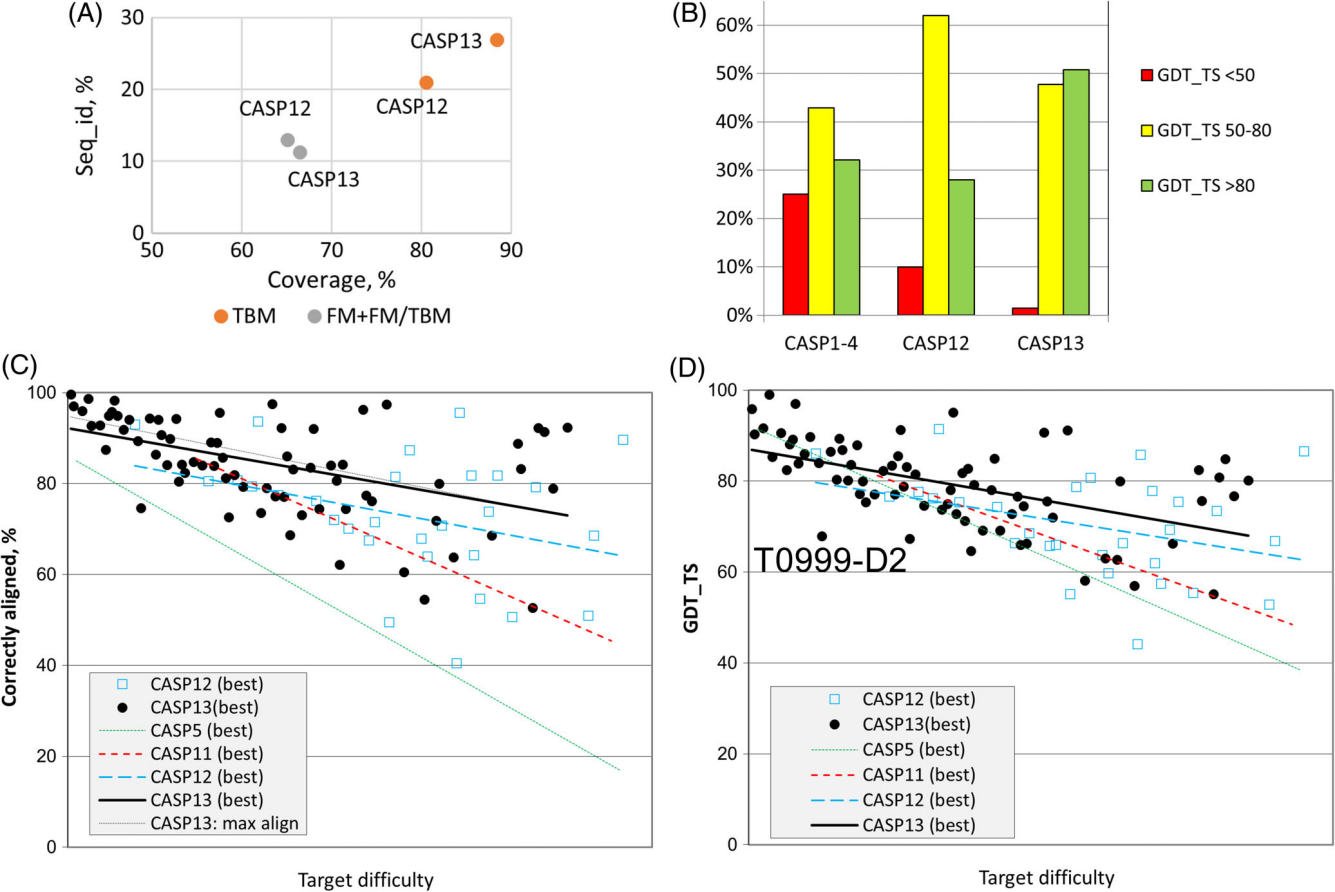
Overall trends in model difficulty and accuracy over time. A, The average difficulty of TBM targets in CASP13 was somewhat lower than in CASP12, with templates of both higher sequence identity and coverage available. B, The distribution of GDT_TS scores for TBM models has shifted toward higher values since the first four rounds of CASP, with a further substantial shift from values below 50 to very good values above 80 between CASP12 and CASP13. C, The accuracy of sequence alignments has improved significantly since CASP11, particularly for low homology templates. D, In keeping with (C), GDT_TS scores appear to still be improving for harder targets. T0999‐D2 is an outlier due to ambiguity in the definition of a “domain,” as discussed in the main text. In (C) and (D), individual data points are shown for CASP12 and −13, with only trend lines shown for earlier meetings. Each point represents the best model submitted by any group for a given target

**Figure 2:**
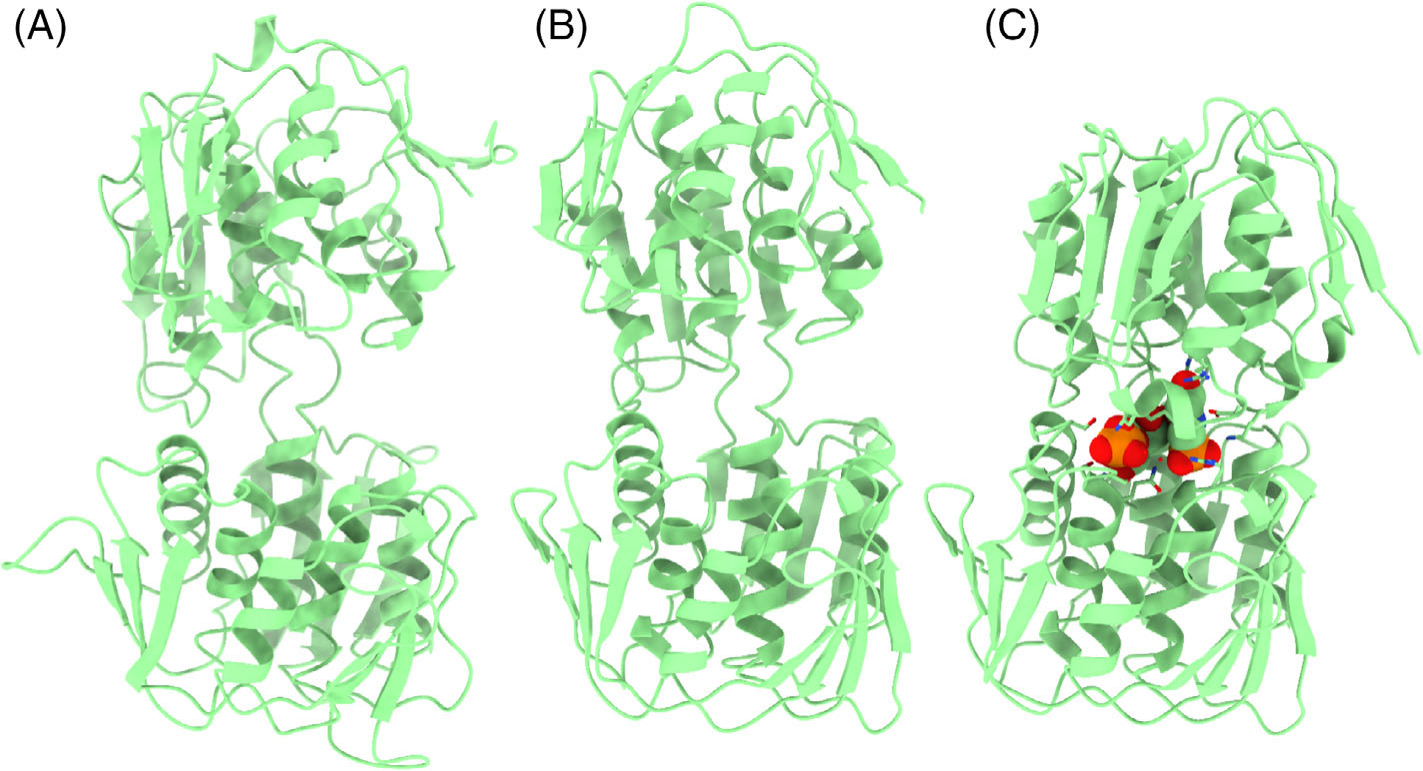
A, T0999‐D2; B, wwPDB entry 5xwb (ligand‐free open conformation^43^); and C, wwPDB entry 3nvs (ligand‐bound closed conformation; ligand shown in space‐filling representation). All three structures are aligned to superimpose the bottom domain. Only models based on an open conformation as in 5xwb will resemble the target. There may be additional flexibility in the open state, as the relative orientations of the domains in T0999‐D2 and 5xwb differ somewhat

With those provisos in mind, a consistent measure of target difficulty can be used to compare results with different targets. In line with previous rounds including CASP12, we use here a linear combination of coverage by the best structural template and the sequence identity between the target and the best template.^5^ Using this measure of difficulty, progress in improving overall fold accuracy as judged by GDT_TS had seemed to stall around the time of CASP11.^38^ However, substantial improvements were seen again in CASP12.^5^ Figure 1 shows that this progress has continued for CASP13. Note that the marked outlier from CASP13 in Figure 1D, T0999‐D2 (a TBM‐hard target), is an example of the challenges involved in designating evaluation units. This target is from a family of proteins that undergo a large conformational change upon ligand binding; since good templates exist in both ligand‐bound and ‐free states, the resulting GDT_TS score for a given model was primarily dependent upon the specific choice of template (Figure 2).

### 3.2 Group rankings

For the primary rankings, predictions were scored by the S_CASP12_ score discussed above. For group rankings, we considered only the scores for the “model 1” models rather than the best of the potential five models submitted for each target. This is the approach generally taken in CASP assessment, and the ability of the group to rank their models forms an implicit part of the ranking score. Any ranking score that assigns comparable weights to a combination of metrics measuring global fold, local fold, and estimated model accuracy is likely to lead to a similar overall ranking, as the metrics within these general categories tend to be highly correlated to one another.^21^ Because the ASE accuracy self‐estimate score measures an orthogonal characteristic of the models (and to assess the possibility that a good ASE score could be attained by assigning large errors to poor models), we also tested the effect on ranking of excluding the ASE measure.

**Figure 3:**
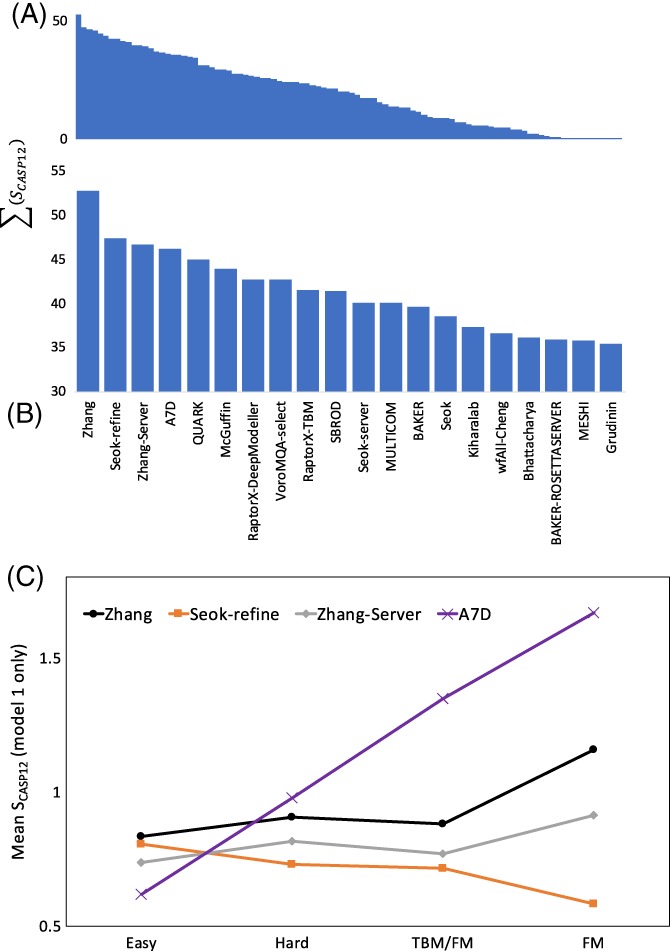
A,B, Overview of TBM rankings for (A) all 99 groups and (B) top 20 groups. Rankings are based on the sum of s_CASP12_ scores for all models designated “model 1” submitted in the TBM‐easy and ‐hard categories. C, Performance across difficulty categories for top four TBM groups. While template‐based methods performed best in the TBM‐easy category, the template‐free machine learning methods of the A7D group clearly outperformed in categories where template homology was weak or nonexistent

Figure 3A shows an overview of the group rankings across all TBM targets, while Figure 3B focuses on the 20 top‐ranked groups. Figure 3C further breaks down the performance of the top four groups as a function of target difficulty (extended to include the TBM/FM and FM categories to give a wider range), and clearly illustrates the usefulness of machine learning methods where available templates are poor or nonexistent (see below).

Three of the top five ranked predictor groups (Zhang, Zhang‐server, and QUARK in positions 1, 3, and 5) are from the same research group. The methods share a step in which a deep multiple sequence alignment is used for contact prediction by a deep neural network, and expected contacts are added to the potential function used in modeling, starting from templates. Seok‐refine (position 2) is a meta‐server that uses quality assessment to choose the best CASP server model (potentially including Zhang‐server and QUARK), which is then further refined. Group A7D (position 4) is unusual in not using explicit template models. Instead, deep multiple sequence alignments are used in a deep convolutional neural network (CNN) to predict a distance histogram for pairs of residues, instead of a binary contact classification, then these form part of a statistical potential trained on the PDB using another deep CNN. Further details are presented in the contribution from the A7D group in this volume. The results echo the conclusion from CASP12 that the introduction of contact prediction was a key advance,^5^ and the introduction of further deep learning algorithms is providing additional power.

Inclusion of the ASE metric in S_CASP12_ is seen to affect the rankings by changing the local ordering, but assigning large estimated errors to inaccurate models does not appear to improve their ranking dramatically. Excluding the ASE metric, the top five groups are A7D > Zhang > MULTICOM > Seok‐refine > McGuffin, which leaves three of the top five groups still in the top five. We believe that it is appropriate to include ASE with a substantial weight, because knowing how confident you should be in what you know is nearly as important as what you do know. As shown below, in the context of MR, this has a practical value in real applications of models.

### 3.3 Server models

Nonexpert users are relatively unlikely to install modeling software locally. Modeling servers are therefore of great importance to a large user community, so it is encouraging to note the good performance of a substantial number of servers in this category. The following server groups are ranked in the top 20 of 99: Zhang‐server (position 3), QUARK (5), RaptorX‐DeepModeller (7), RaptorX‐TBM (9), Seok‐server (11), and BAKER‐ROSETTASERVER (19).

### 3.4 Geometric model quality

The S_CASP12_ score is based primarily on global (GDT_HA) or local (lDDT, SG, CADaa) Cartesian distance‐based metrics. While useful for assessing the match of a given model to the overall fold of a domain, it was not clear to us whether these are sufficient to distinguish between models for which the predicted fold is essentially correct. Beyond this point, the next most important challenge is arguably matching the fine details—that is, the disposition of each residue's peptide bond and (where applicable) sidechain atoms.

As a complement to the standard scoring metrics, for each individual model submitted we therefore assessed the conformational similarity to the target on a per‐residue basis. Specifically, for each residue present in both model and target we computed two scores: S_backbone_ based on the average error in the three diagnostic backbone torsions *φ, ψ*, and *ω*, and S_sidechain_ based on errors in the first two sidechain *χ* torsions. We also recorded common serious errors revealed in the *ω* torsion, namely *cis*/*trans* peptide bond isomer disagreement, and peptide bonds twisted more than 30° out of plane. While *cis* peptide bonds are rare (found in approx. 5% of proline residues and 0.03% of non‐prolines), stable twists of more than 30° can be considered essentially impossible, having almost never been observed in experimental structures.^27^ If a model named one or more template(s), we analyzed the template most closely corresponding to the target domain in the same manner, to assess if and where the modeling improved the result.

**Figure 4:**
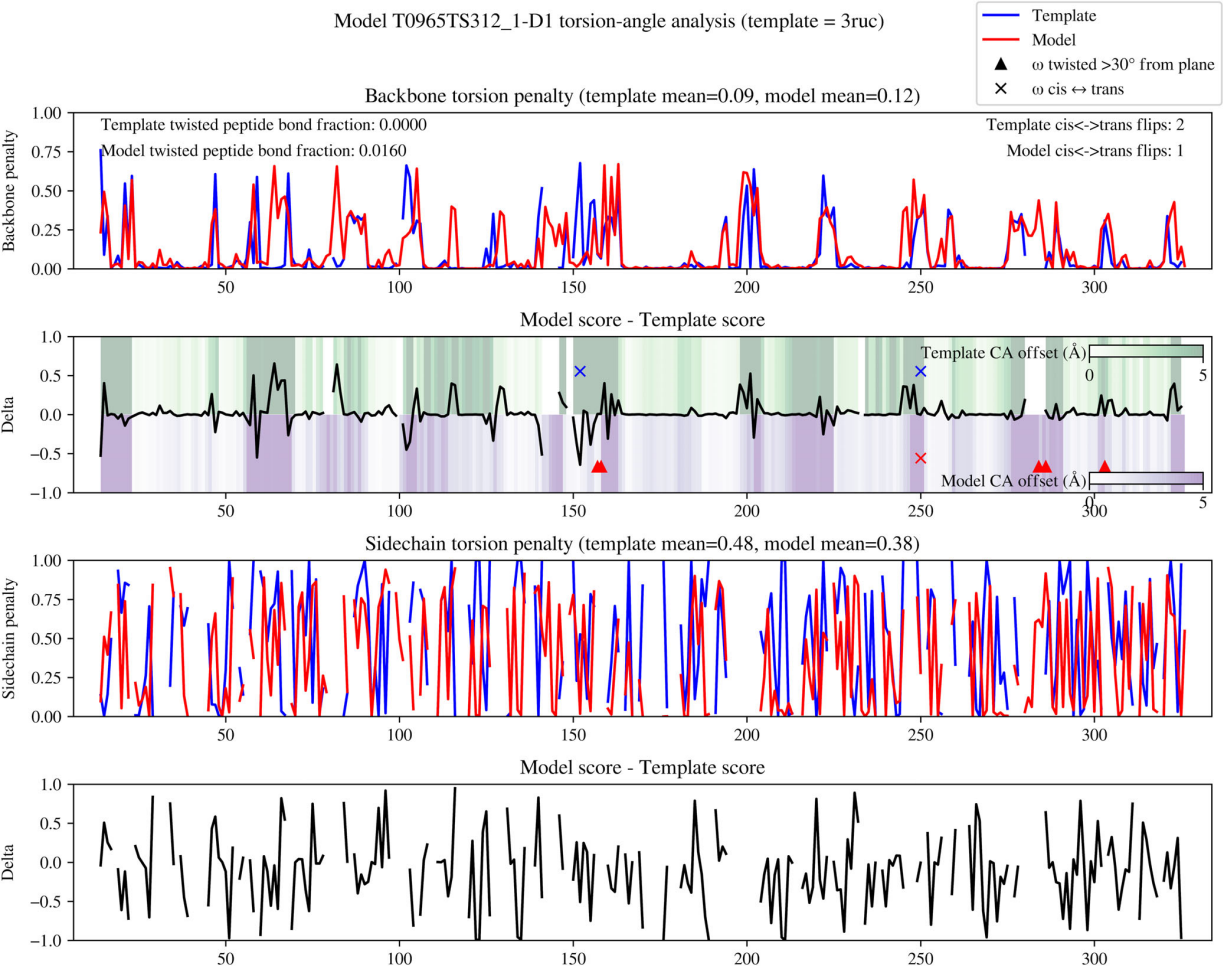
Example summary chart from torsion‐space comparison of template and model to target for T0965‐D1 (TBM‐hard). Top panel: per‐residue backbone torsion deviations from the target (lower is better). Second panel: difference between template and model results from top panel—negative values indicate the model has improved agreement compared to the template. Background coloring indicates the residual differences in Cα positions between template (green) or model (purple) and target after rigid‐body alignment. Sites with potentially problematic peptide bonds (cis/trans disagreement or twisted more than 30° from planar) are indicated with crosses and triangles respectively. Third panel: sidechain dihedral errors, weighted for degree of burial and distance from backbone as described in the main text. Bottom panel: difference between template and model sidechain results—negative indicates improvement

An example summary chart for a model in the TBM‐hard category is shown in Figure 4. A particularly notable feature of this particular case is the region around residue 150: here the modeling has corrected a *cis*/*trans* disagreement between template and target, while significantly increasing both the backbone conformational agreement and Cα positioning of the surrounding eight residues. On the other hand, this comes at the expense of two severely twisted peptide bonds nearby.

It is clear that modeling of sidechains onto scaffolds based on distant homology remains a significant challenge. The sidechain score for this model (0.383) is very close to the mean of 0.367 for all models submitted for this target. In contrast, the A7D group (who, as detailed elsewhere in this volume, eschewed all template‐based information in favor of using ROSETTA^39^ to build energetically favorable sidechains onto a folded poly‐Gly scaffold) achieved the lowest sidechain score for this target (0.241). On the other hand, in the presence of strong homology, sidechain information in the template is clearly far more useful. In TBM‐easy target T0961‐D1, for example, the best model from A7D (0.217) is only slightly better than the all‐model average (0.247), and significantly worse than the best performing model (Kiharalab, 0.156).

**Figure 5:**
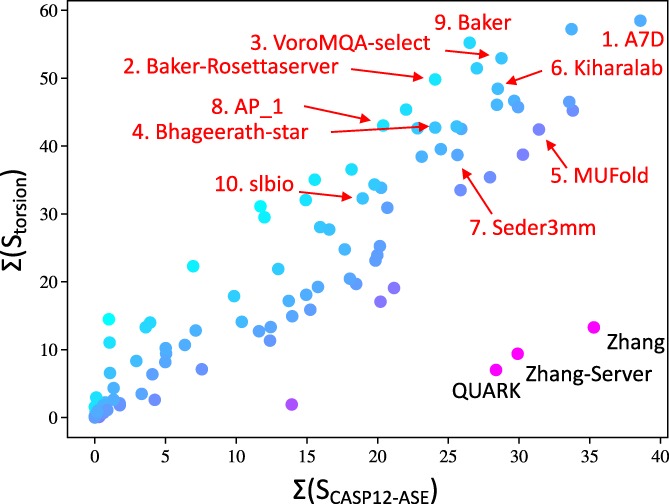
Torsion‐based scoring metrics reveal issues not captured by standard scores. Horizontal axis: sum of all positive *z*‐scores by standard ranking formula. Vertical axis: sum of all positive *z*‐scores by torsion‐only formula. Each point represents the aggregate of all models submitted by a single group in the TBM‐easy and TBM‐hard categories. Points are colored according to change in ranking going from S_CASP12‐ASE_ to S_torsion_. The top 10 groups in molecular replacement trials disregarding error estimates (see Figure 7A) are marked in red. The three points at lower‐right (each originating from I‐TASSER^15^) demonstrate that it is possible to achieve excellent (indeed, field‐leading) scores by default metrics while still suffering from severe distortions at the local level

In order to compare the results of this analysis we derived a “torsion‐only” ranking score S_torsion_ based on a weighted combination of backbone and sidechain errors (see section 2). Comparing this to the S_CASP12‐ASE_ score aggregated over all models (Figure 5) revealed that high TBM scores are no guarantee of good local geometry—indeed, the otherwise field‐leading contributions from the Zhang lab^15^ score in the bottom quintile of all groups by the torsion‐only metric.

It is also interesting to note that neither score correlates particularly well with the suitability of models for MR (discussed below). While the top 10 groups by this MR score (highlighted in red) are found for the most part in the upper‐right quadrant of the plot, they share this space with a much larger number of similarly scoring groups whose models were not as effective for MR.

**Figure 6:**
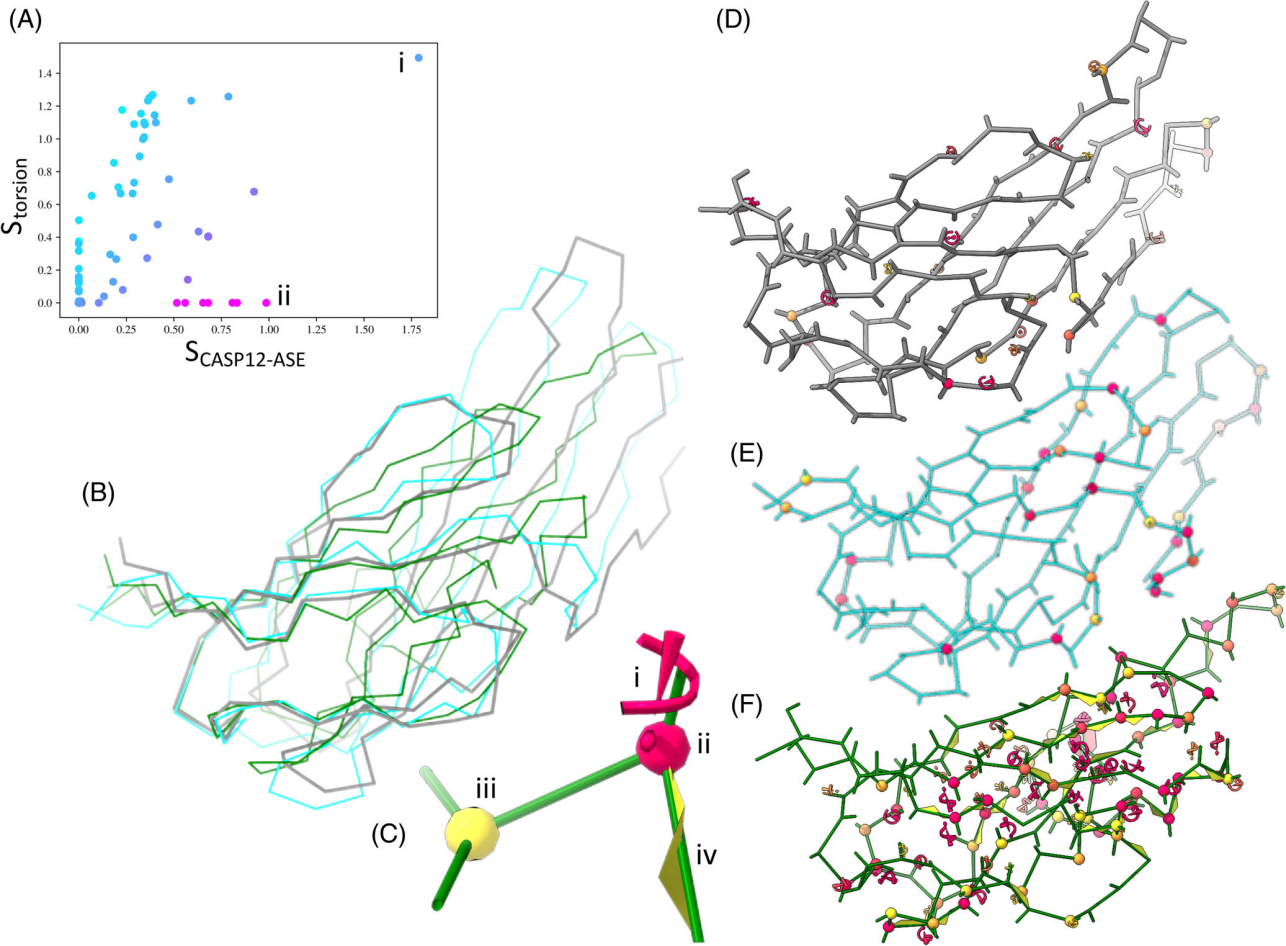
Target T0981‐D5 (TBM‐hard) presents a particularly stark example of the importance of carefully considering model stereochemistry. A, The two leading models by S_CASP12‐ASE_ (horizontal axis) (i: A7D; ii: Zhang‐Server) appear at opposite extremes according to S_torsion_ (vertical axis). Note: the corresponding S_CASP12_ scores (including the ASE measure) for these two models are 1.79 and 1.55, respectively. B, The Cα correspondence to the target is quite similar in both cases: close in the core fold while deviating substantially on the two extended hairpins at right. Gray = target; cyan = A7D; green = Zhang‐Server. C, Summary of markup used in panels D through F. (i) Severe sidechain outlier (*P* < .05%). Less severe outliers appear as smaller, yellow‐orange versions of the same motif. (ii, iii) Ramachandran outlier (*P* < .05%) and marginal (*P* < 2%) respectively. (iv) Peptide bond twisted more than 30° out of plane. D‐F, While the A7D model (E) contains a similar number of Ramachandran outliers to the target (D), more than half of all residues in the model from Zhang‐Server (F) contain Ramachandran, sidechain and/or peptide bond planarity outliers

The disparity between Cartesian and torsional scoring metrics is illustrated further in Figure 6 using T0981‐D5 (TBM‐hard) as an example. By S_CASP12_ it appeared that A7D and Zhang‐Server did comparably well on this model, with *z*‐scores of 1.76 and 1.55 respectively. By S_CASP12‐ASE_ these remained the leading models, but the gap was markedly widened (*z*‐scores of 1.77 and 0.98 respectively). In torsion space, however, the A7D model remained the leader (*z* = 1.49; S_backbone_ = 0.123; S_sidechain_ = 0.234) while the Zhang‐Server model received the minimum possible *z* score (*z* = 0; S_backbone_ = 0.211; S_sidechain_ = 0.370). Inspecting more closely revealed that many sites in the latter were not simply incorrect relative to the target but were physically highly implausible: 36 (28%) of peptide bonds were either twisted >30° from planar or flipped into *cis* conformation; 33 sidechains (26%) were rotamer outliers; and 65 residues (51%) were outside of favored Ramachandran space.

### 3.5 MR model quality

**Figure 7:**
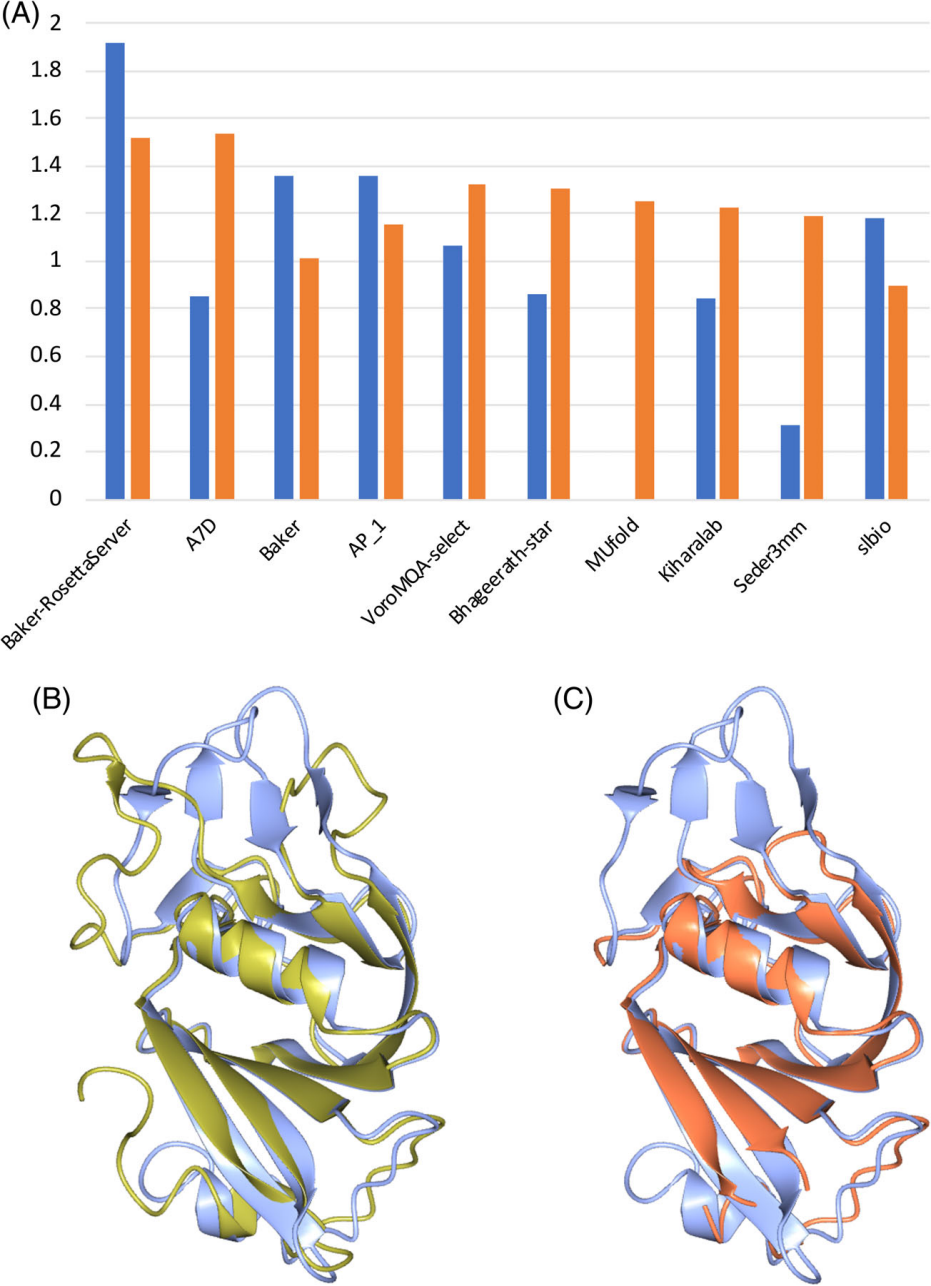
A, Top 10 groups ranked by mean *z*‐scores for LLG calculations. Groups are sorted by the maximum of the mean *z*‐score computed using the calculations where the B‐factor column is interpreted as an RMS coordinate error estimate for each atom (blue bars) or where constant B‐factors are used (orange bars). (B,C) Effect of B‐factor weighing on MR utility for BAKER‐ROSETTASERVER model of T1002‐D3. Both panels show the experimental structure of T1002‐D3 in blue. Panel (B) shows the best model (number 3) submitted by BAKER‐ROSETTASERVER in gold. Panel (C) shows the same model in salmon, but only including the residues for which the estimated coordinate error was less than 2 å

Suitable diffraction data were available for 20 of the TBM target structures, which contributed 27 of the 61 evaluation units in this category. Groups were ranked by mean LLG *z*‐score, choosing the best model for each target. As shown in Figure 7A, the very best results were achieved for models that were accompanied by good error estimates, although not all groups provided error estimates that improved the utility of their models for MR. These results provide a concrete illustration of the concept that it is just as important to know how accurate your predictions are as to have accurate predictions in the first place. For instance, the models from group A7D on the whole gave the highest LLG scores without error weighting, by a narrow margin, but with error weighting the BAKER‐ROSETTASERVER models were significantly more useful. (As discussed below, problems with the error estimates from group A7D probably arose from ambiguity in whether the error estimates were meant to apply to the complete structure or just within an evaluation unit.)

One of the best examples of the utility of error weighting for MR comes from target T1002‐D3 (TBM‐easy). For this target, the best model using constant B‐factors is model 2 from the YASARA group, yielding an increase of 1053 in LLG from the background. By comparison, model 3 from the BAKER‐ROSETTASERVER group yields an increase in LLG of 989 when evaluated with constant B‐factors, which increases dramatically to 3186 when B‐factor weighting is applied. Panels b and c of Figure 7 illustrate the success with which the unreliable parts of the structure have been identified by assigning large estimated errors.

Results from the group Seder3mm reveal a complication in evaluating the effect of changes to the B‐factor used in the MR calculations. The perfect model would be one in which both the coordinates and the B‐factors are correct. For a model with errors in the coordinates, the optimal B‐factors will be ones in which the correct B‐factor is inflated to compensate for the coordinate error, thus smearing the density of the atom over the correct position. Predictors are not asked to predict the actual B‐factors, so the only clear choices for evaluation are to use constant B‐factors or B‐factors derived from the predicted coordinate errors. Nonetheless, LLG values were computed as a control interpreting the B‐factor columns as B‐factors, and the results for Seder3mm were extreme outliers in this calculation, yielding a mean *z*‐score of 1.38. This would have placed the group in third place in Figure 7A, rather than ninth place. The improvement apparently comes not from predicting errors but rather from predicting the B‐factors themselves, using a formula based on depth of burial of a residue and an entropy factor computed taking account of the secondary structure (Eshel Faraggi, personal communication).

A comparison of Figure 7A with Figure 3 reinforces the impression from Figure 5 that the ranking by utility for MR is very different from the one obtained with S_CASP12_. As discussed in the evaluation for high‐accuracy TBM in CASP7,^6^ utility for MR depends on a substantial fraction of all atoms being placed reasonably accurately, but once an atom is far from the correct position, a larger error will not reduce the score further. In contrast, the more conventional measures focus on the trace of the fold, and penalties increase as errors increase.

**Figure 8:**
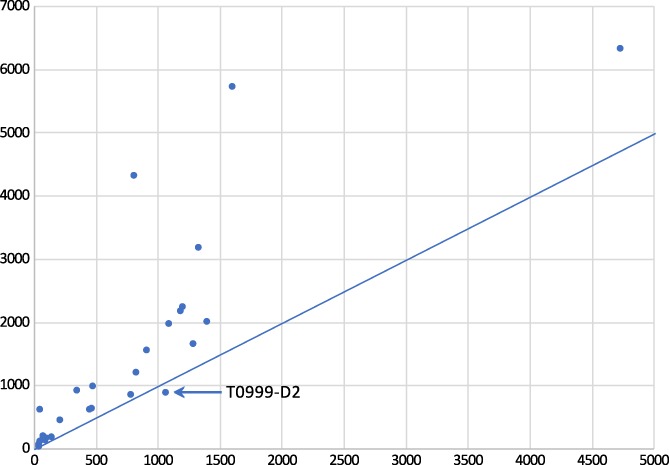
Value added for utility in MR. For 26 of 27 evaluation units, the best model is better than the best template previously available from the PDB

Finally, the results show that substantial progress has been made since CASP7, when this metric was last used in assessing the TBM category. At that time, the very best model improved on the best template for only 5 of 12 evaluation units. This time, the best model improves on the best template for 26 of the 27 evaluation units (Figure 8). The single exception is T0999‐D2 (TBM‐hard) for which, as discussed above, the relative orientation of two domains is uncertain in the absence of knowledge about ligand‐binding state. In fact, the bar was set higher in this evaluation, because ensemble models were included among the templates used for comparison. In nine cases, the best template model was actually an ensemble model.

## 4 DISCUSSION

### 4.1 Error self‐assessment requires clearer criteria

The intention when requesting the estimated coordinate error was for it to be an estimate of *local* error—in essence, what would be the error of this atom after rigid‐body alignment of its local domain (the evaluation unit) to the target? However, we now realize that this has not been unambiguously communicated to participants, the instructions provided on the CASP website being simply, “In place of temperature factor field, the error estimates, in ångstroms, should be provided.” Nevertheless, the majority of groups applying template‐based methods appear to have used the desired interpretation—probably because this form of error estimate is the most natural for this approach. However, an equally valid interpretation is, “how sure are we of the coordinates of this atom relative to the *entire chain*?” While identical to the former interpretation for single‐domain proteins, this yields vastly different results in the case of large, multi‐domain chains where the relative domain orientation is uncertain. In this round, group A7D (quite reasonably) applied this latter interpretation, leading to error estimates that were in many cases 1‐2 orders of magnitude larger than expected. For future CASP rounds, the definition of coordinate error estimates should be more carefully specified.

### 4.2 Geometry‐weighted scoring metric

As we have shown, under the current‐standard S_CASP12_ scheme it is possible to generate a top‐scoring model that nevertheless contains a very large number of physically implausible or impossible local conformational features. It thus seems reasonable to suggest an alternative scoring scheme incorporating these. The weighting of these must be carefully considered, however. Relying purely on torsions is inadvisable: as a simple illustration, for a target consisting of a bundle of alpha helices a model built as a single long helix will score almost as well as the correct fold by this metric. It is also wise to consider that the experimental structure itself is not perfect, and that torsions (particularly in elements without defined secondary structure) tend to be substantially more error‐prone than Cα and overall sidechain positions. On the other hand, inclusion of torsion‐based scoring will reduce the effect of the (not uncommon) case where some portion of the target is likely flexible in solution but has been captured in a single conformation. In such cases, distance‐based metrics will unfairly reward models which happen by chance to replicate the specific location of the flexible element, whereas torsion‐based metrics remain largely unaffected.

**Figure 9:**
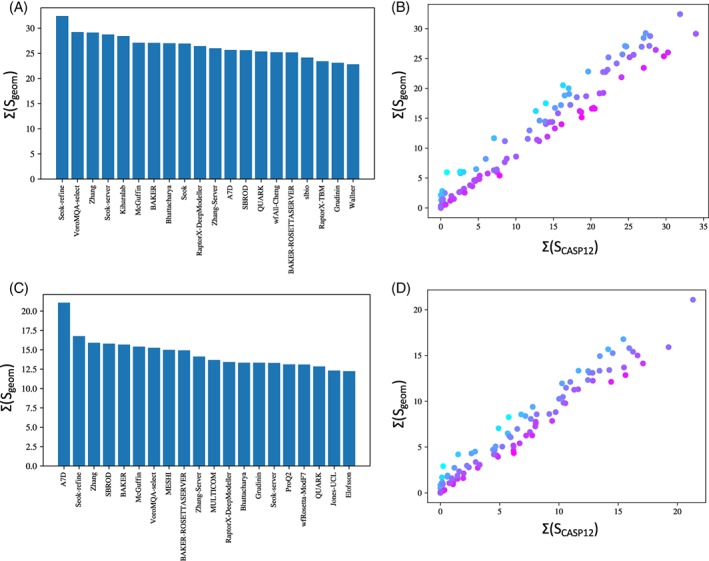
Rankings by geometric quality for (A,B) TBM‐easy and (C,D) TBM‐hard categories. A,C, Scores for top 20 groups in each category. B,D, comparison of S_geom_ vs the standard S_CASP12_. It is particularly notable that A7D, the top group in TBM‐hard—by either metric—did not in fact use a template‐based method

Here we describe one possible such scheme, S_geom_, combining the existing ASE, GDT_HA and local distance‐based methods (lDDT, SG, and CADaa), backbone and sidechain torsion errors, and the MolProbity clashscore (a strong determinant of general model quality which is not captured by other metrics). Re‐rankings according to this scheme are shown in Figure 9.

### 4.3 Care is needed when choosing templates

The models deposited in the wwPDB cover an extraordinarily wide range of both resolution and quality, from essentially‐perfect structures built into sub‐ångstrom resolution maps to ones that are essentially just homology models rigid‐body docked into domain‐scale “blobs”. Between these extremes factors such as variations in data quality, ability of the practitioner, and the progressive improvement in software over time can mean that even models of comparable resolution may have dramatically different stereochemical quality.^40^ One measure of stereochemical quality widely used in experimental structural biology is the MolProbity score,^27^ a single log‐scale value summarizing unfavorable backbone/sidechain conformations and the number of badly‐overlapping nonbonded atoms. A score less than 1.5 typically indicates a “good” model (ie, of a quality that one would expect from atomic‐resolution data) while scores greater than about 2.5 are cause for caution. The maximum possible MolProbity score (for a hypothetical model where every atom is clashing, and every residue is both a Ramachandran and rotamer outlier) is approximately 6.1. The Ramachandran, rotamer, and clashscore statistics necessary to calculate a MolProbity score may be conveniently parsed from the XML‐format validation files provided on the wwPDB FTP server, avoiding the need for computationally intensive reanalysis.

In this CASP round, 1651 individual models were identified as templates in the submitted model files. Of these, 15 could not be retrieved from the wwPDB—11 due to having been obsoleted and replaced with newer models, one withdrawn entirely, one apparently nonexistent and two unreleased at the time of writing. Model 1wb1, for example, was used as a template for target 1022 by five groups, despite having been obsoleted and replaced by 4ac9 more than 5 years before the beginning of this CASP round. This is a problem that is likely to be compounded in future by the (otherwise positive) recent decision by the wwPDB steering committee to begin allowing authors to deposit updated versions of coordinates under their original accession ID: given that the wwPDB is a rapidly‐growing and ever‐more‐dynamic database, any static template library is doomed to quickly become outdated.

**Figure 10:**
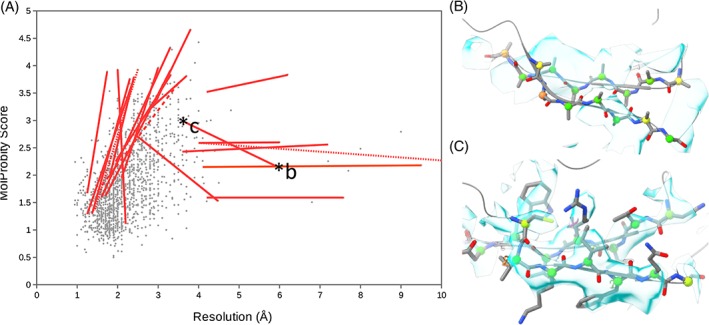
Importance of considering model quality when selecting templates. A, Scatter plot of resolution vs MolProbity score for all PDB entries identified as templates used in this CASP round (excluding models with resolutions below 10 å). Red lines connect selected templates to similar models with significantly better resolution and/or MolProbity score. Alternative models were selected from those with better than 90% (solid lines), 70% (dashed line), or 50% (dotted lines) sequence identity to the template chain. B, Representative fragment of chain F from 5mqf (5.9 å resolution cryo‐EM model used as a template for T0954 by six groups). The density is uninterpretable on the atomic scale—this chain is a homology model, truncated to poly‐Ala and rigid‐body docked into patchy density. C, Equivalent region from the 100% sequence‐identical 5xjc (3.6 å cryo‐EM model, used by only two groups). All sidechains are present and for the most part modeled into strong, convincing density. The lower MolProbity score for 5mqf arises simply because truncated sidechains do not contribute to clashscore nor count as rotamer outliers

Nine templates were of resolutions lower than 10 å. For the remainder that could be retrieved from the wwPDB, a plot of MolProbity score vs resolution is shown in Figure 10. For the most extreme low‐resolution and/or low‐quality templates in this cohort, we searched for better models of similar or identical sequence with release dates of 2017 or earlier (ie, those that were available for this CASP round). Of the approximately 50 cases inspected, we were able to find demonstrably better models for 21 (Table 1). Eighteen of these were >90% identical in sequence to the template, and 12 were never used as templates by any group. In Figure 10A, red lines connect each template to the identified alternative.

**Table 1:** Details of alternative templates for the cases pictured in Figure 10A

Template (alternative)	Identity	Resolution å	Clashscore /1000	Rama outliers %	Rotamer outliers %	MolProbity score
5l5g_A (5l56)	>50%	10 (4.0)	29.3 (10.1)	0.96 (1.20)	1.14 (5.12)	2.27 (2.59)
3j2t_A (5juy)	>90%	9.5 (4.1)	15.9 (15.6)	2.09 (0.45)	0.81 (0.71)	2.18 (2.15)
4kss_A (4ksr)	>90%	7.58 (4.2)	5.52 (4.47)	0.39 (0.46)	1.05 (1.28)	1.59 (1.59)
5nrl_H (5gap)	>90%	7.2 (3.6)	22.6 (7.13)	0.32 (1.47)	0.19 (4.23)	2.56 (2.43)
5li2_A (5li4)	>90%	6.2 (4.2)	163 (92.1)	5.32 (7.11)	3.58 (2.75)	3.83 (3.52)
5l59_A (5l56)	>90%	6.0 (4.0)	13.3 (10.1)	1.34 (1.20)	3.74 (5.12)	2.6 (2.59)
5mqf_F (5xjc)	>90%	5.9 (3.6)	11.0^a^ (23.6)	0.81 (1.82)	0^a^ (8.13)	2.17 (2.99)
4rvw_A (4rdr)	>90%	4.48 (2.47)	1.36 (15.32)	0.33 (0.14)	1.79 (5.94)	1.53 (2.72)
2qfi_A (3h90)	>90%	3.8 (2.9)	118 (13.4)	27.1 (2.85)	30.5 (20.41)	4.66 (3.28)
2etn_A (2f23)	>90%	3.3 (1.6)	68.7 (12.33)	12.4 (0.00)	35.1 (0.77)	4.33 (1.60)
1bgy_D (2a06)	>90%	3.0 (2.1)	61.3 (11.3)	4.0 (0.66)	19.3 (1.62)	3.96 (1.95)
1ubv_A (4nua)	>50%	2.5 (1.43)	46.9 (4.03)	5.2 (0.00)	24.1 (1.62)	3.93 (1.35)
1msc_A (4zor)	>90%	2 (2.2)	44.1 (2.31)	12.6 (0.16)	21.7 (1.34)	3.93 (1.11)
3s4d_A (3rrs)	>90%	3.3 (1.7)	83.0 (4.25)	1.10 (0.00)	15.7 (1.45)	3.91 (1.62)
4hhb_D (2dn2)	>90%	1.74 (1.25)	141 (4.64)	1.24 (0.00)	8.7 (3.7)	3.89 (1.67)
1nqg_A (2guf)	>90%	3.31 (1.95)	64.6 (11.1)	3.7 (0.37)	17.3 (8.0)	3.83 (2.25)
2ziy_A (4ww3)	>90%	3.7 (2.8)	50.5 (28.9)	8.4 (2.3)	9.7 (7.1)	3.81 (3.21)
1g59_A (1j09)	>90%	2.4 (1.8)	46.3 (10.5)	1.72 (0.21)	26.9 (3.6)	3.79 (1.96)
1dcl_A (5wca)	>90%	2.3 (1.37)	47.5 (3.1)	4.4 (0.46)	19.6 (1.34)	3.76 (1.30)
1unx_A (4hjb)	>90%	2.4 (1.25)	30.4 (5.4)	4.9 (0.00)	33.9 (0.00)	3.76 (1.29)
4ih4_A (3w04)	>70%	3.5 (1.45)	42.0 (7.2)	7.5 (0.00)	10.1 (1.76)	3.68 (1.65)

a 5mqf is a poly‐alanine model only, causing rotamer and clashscore statistics to become misleading.

Our observations here suggest that an easy way for some groups to significantly improve their TBM results will be to introduce some simple extra heuristics in the selection of templates. Our recommendations are:

- Only use templates with resolution poorer than 5 å with extreme caution, and if no other option exists. At these resolutions the model is likely to be little more than a Cα trace and/or set of rigid‐body fitted homology domains (see for example Figure 10B). Sidechains are effectively invisible, and the backbone path is typically extremely vague with the possible exception of long helices. Replace with a higher‐resolution template wherever possible, even at the expense of significantly lower sequence homology.
- Resolution 3.5‐5 å: while most of the fold is typically correct at these resolutions, it is common for stretches of up to a few dozen residues to be out of register (ie, systematically shifted one or more positions toward their *N*‐ or *C*‐terminus). MolProbity scores higher than ~3 are cause for substantial caution. If an alternative exists with higher resolution but lower sequence homology, consider using that instead. Favor models with complete sidechains over those with truncated ones.
- Resolution 2.5‐3.5 å: a “transition zone” where most of the model is *usually* correct. Most sidechains are at least partially visible and regions with defined secondary structure will usually be well‐modeled, but loop regions are often problematic.
- Resolution <2.5 å: except in very rare cases (or very old models) these are generally trustworthy.
- Favor newer models over older ones—data collection, computational methods, and validation statistics have all improved dramatically, particularly over the past 15 years. The two oldest models used in this round were 2hhb and 4hhb, two models of human deoxyhemoglobin deposited in 1984; 209 newer, and 28 higher‐resolution, experimental models of this protein exist.
- All else being equal, choose the model with the highest resolution, followed by the lowest MolProbity score. Wherever possible, avoid models with MolProbity scores greater than about 3 and ideally aim for those with scores below 2. Developers might also consider using properties at the residue level such as difference from the mean B‐factor for that structure (a useful proxy for local effective resolution and/or coordinate error), Ramachandran and rotamer probabilities, and local clashes to assign finer‐grained confidence scores to templates.
- Reduce or remove reliance on static template libraries in favor of selection directly from the wwPDB. Software and server developers should consider making use of the extensive query APIs provided by the RCSB PDB^41^ and/or PDBe^42^ to select up‐to‐date templates directly from the master wwPDB database.

### 4.4 MR score could inspire a more general metric

The MR LLG score has a substantial potential advantage in providing a numerical measure of the utility of a model for one of the purposes to which models are put, that is, solving new crystal structures. It assesses not only a measure of all‐atom accuracy but also provides a tangible reward for good estimates of coordinate accuracy.

However, there are serious drawbacks to this metric as it stands. The most obvious is that it can only be used when diffraction data have been made available. Even when diffraction data are available, the scores for models of different targets are not on the same scale, because the LLG values depend on the number of reflections in the data set, and the sensitivity to model errors depends on the resolution to which the data extend. When targets are subdivided into evaluation units, different fractions of the full structure will provide the background for the LLG calculation covering an evaluation unit; because of the quadratic dependence of the LLG on the completeness of the model,^32^ the calculations will all be on different scales. Data pathologies such as anisotropic diffraction or tNCS add further complications.

What is needed is a metric that reflects utility in MR but can be calculated on the same scale for any evaluation unit. Such a metric could be based on the correlation of electron densities between the model and the target, determined in shells of resolution. These correlations are closely related to the *σ*_A_ values used in the likelihood calculations, from which we can infer that the LLG should be proportional to an integral over resolution of the fourth power of the electron density correlation (deduced from the functional form of the expected LLG calculation^32^), weighted by the square of the inverse resolution (to account for the density of Fourier terms as a function of resolution). In future work, we hope to implement and test such a metric. Because both crystallography and cryo‐EM methods involve fitting 3D atomic models to maps, either by correlating their electron density (X‐ray crystallography) or their electric potential (cryo‐EM), this metric would be highly useful in optimizing modeling procedures that assist in experimental structure determination.

## 5 CONCLUSIONS

The progress in the TBM category that was seen in CASP12 has continued in CASP13. As noted for CASP12, the use of contact (or inter‐residue distance) predictions derived from deep multiple sequence alignments is an important ingredient. One surprise, found in the work from the group A7D that used deep convolutional neural networks, is that explicit use of template models is not essential, though it is still valuable when there are closely related templates available in the wwPDB.

We were also surprised to find that few predictors appear to be taking account of measures of experimental structure reliability when choosing templates to use as starting models (or presumably to train structure prediction methods in general). Applying some simple rules when choosing templates should have an immediate impact on model quality.

Given the progress that has been achieved in the TBM category since the inception of the CASP experiments, we believe that this is a good time to raise the level of expectations for good quality models. The results from some predictors show that it is possible not only to predict the general outline of a protein fold but also to predict more of the details in terms of the main‐chain and side‐chain torsion angles, as well as to evaluate the local reliability of their models. To encourage development along these lines, we present a new suggested ranking score that future assessors might wish to consider as a basis for their work.

## CONFLICT OF INTEREST

The authors have no conflict of interest to declare.

## Supporting information


**Appendix S1:** Supporting Information
