# Supplementary material for: Evaluation of template‐based modeling in CASP13
Source: Proteins. 2019 Aug 20;87(12):1113–27. doi: 10.1002/prot.25800 (PMC6851432; doi:10.1002/prot.25800)
Supplement: Supplementary file 1 — Appendix S1: Supporting Information [file PROT-87-1113-s001.pdf]

Supplementary methods

The original version of this manuscript prior to peer review used the following scoring functions for geometric quality.

For each residue present in both model or template and target, the backbone score was defined as:

$$S_{backbone} = \frac{|\Delta\varphi| + |\Delta\psi| + |\Delta\omega|}{3}$$

where φ and ψ are the characteristic Ramachandran torsion angles and ω is the torsion across the peptide bond.

The sidechain score was defined as:

$$S_{sidechain} = \beta\left(\sum_{i=1}^{n_\chi}\frac{\Delta\chi_i}{2^i}\bigg/\sum_{i=1}^{n_\chi}\frac{1}{2^i}\right)$$

where χ_i is the ith sidechain torsion from the backbone, and β is a “burial score” defined as:

$$\beta = \min\left(\frac{n_{close}}{3n_{sc}}, 1\right)$$

where n_{close} is the number of heavy atoms from other residues within 4Å of any heavy atom in the given residue (based on the target structure), and n_{sc} is the number of heavy atoms expected in the sidechain. Sidechains with no χ torsions in the target (i.e. glycine or alanine residues and truncations) did not receive a score. For any χ torsions present in the target but not in the model (e.g. not modeled by the depositor, or mutated in the case of a template structure), $\Delta\chi$ was given the maximum possible value of 180°. Any torsions present in the model but not in the target were ignored.
